# NHA1 is a cation/proton antiporter essential for the water-conserving functions of the rectal complex in *Tribolium castaneum*

**DOI:** 10.1073/pnas.2217084120

**Published:** 2023-03-21

**Authors:** Muhammad T. Naseem, Robin Beaven, Takashi Koyama, Sehrish Naz, Sheng-yuan Su, David P. Leader, Dan A. Klaerke, Kirstine Calloe, Barry Denholm, Kenneth V. Halberg

**Affiliations:** ^a^Department of Biology, Section for Cell and Neurobiology, University of Copenhagen, Copenhagen DK-2100, Denmark; ^b^Deanery of Biomedical Sciences, College of Medicine and Veterinary Medicine, University of Edinburgh, Edinburgh EH8 9AG, UK; ^c^Institute of Molecular, Cell and Systems Biology, College of Medical, Veterinary and Life Sciences, University of Glasgow, Glasgow G12 8QQ, UK; ^d^Department of Veterinary and Animal Sciences, Section for Pathobiological Sciences, University of Copenhagen DK-1870, Frederiksberg, Denmark

**Keywords:** rectal complex, *Tribolium castaneum*, secondary cell, cation/H^+^ antiporter, genetics

## Abstract

More than half of all extant metazoan species on earth are insects. The evolutionary success of insects is linked with their ability to osmoregulate, suggesting that they have evolved unique physiological mechanisms to maintain water balance. In beetles (Coleoptera)—the largest group of insects—a specialized rectal (“cryptonephridial”) complex has evolved that recovers water from the rectum destined for excretion and recycles it back to the body. However, the molecular mechanisms underpinning the remarkable water-conserving functions of this system are unknown. Here, we introduce a transcriptomic resource, BeetleAtlas.org, for the exceptionally desiccation-tolerant red flour beetle *Tribolium castaneum,* and demonstrate its utility by identifying a cation/H^+^ antiporter (NHA1) that is enriched and functionally significant in the *Tribolium* rectal complex. NHA1 localizes exclusively to a specialized cell type, the leptophragmata, in the distal region of the Malpighian tubules associated with the rectal complex. Computational modeling and electrophysiological characterization in *Xenopus oocytes* show that NHA1 acts as an electroneutral K^+^/H^+^ antiporter. Furthermore, genetic silencing of *Nha1* dramatically increases excretory water loss and reduces organismal survival during desiccation stress, implying that NHA1 activity is essential for maintaining systemic water balance. Finally, we show that Tiptop, a conserved transcription factor, regulates NHA1 expression in leptophragmata and controls leptophragmata maturation, illuminating the developmental mechanism that establishes the functions of this cell. Together, our work provides insights into the molecular architecture underpinning the function of one of the most powerful water-conserving mechanisms in nature, the beetle rectal complex.

## Significance

Beetles are the largest group of animals on earth. Unique adaptations in overcoming water stress is critical to their success in arid areas, yet the mechanisms underpinning this ability are unknown. Using genetics and electrophysiological studies, we show that a cation/H^+^ (NHA1) transporter is exclusively localized to specialized leptophragmata cells in the Malpighian tubules associated with the rectal complex. Ion transport by NHA1 in leptophragmata underpins the movement of water from the rectum to recycle it back to the body, and is essential for maintaining systemic water balance in beetles. This work provides insights into the molecular architecture of one of the most powerful water-conservation mechanisms in biology, and provides an important clue to the evolutionary success of the beetles.

Insects are (in terms of species) the most diverse animal group on the planet, occupying the widest possible range of habitats on earth (1). However, insects—being a predominantly terrestrial group—are faced with major problems in maintaining ion and water balance as their small size and large surface-to-volume ratio make them highly sensitive to osmotic disturbances. As such, the evolutionary success of insects is intrinsically linked with their ability to defend against harmful changes in their water contents in a wide range of environments. In insects, the renal (Malpighian) tubules (MTs) and the hindgut are the principal organs responsible for regulating body fluid composition (2). Whereas the MTs secrete excess ions and water by producing a primary urine that is drained into the alimentary canal (3), the hindgut selectively reabsorbs solutes and water in proportion to the needs of the animal (4). The hindgut (in particular the rectum) is thus the major site of water conservation in insects as it provides vital feedback control of the final composition and volume of the excretory products. However, surprisingly little is known about the mechanisms that mediate the selective absorption of water and solutes by the rectum in different external conditions.

The physiological importance of the rectum in maintaining overall water balance in insects is particularly evident in species colonizing arid environments. For example, in the mealworm *Tenebrio molitor*—a species that is capable of completing its entire life cycles without access to environmental water—a specialized rectal (“cryptonephridial”) complex has evolved that enables recovery of almost all water from the rectum (5, 6). The anatomical arrangement of this complex is defined by the distal ends of the MTs (perirectal tubules, PTs) being closely applied to the rectal wall with the entire structure enclosed beneath an impermeable perinephric membrane (6, 7). This cryptonephridial condition is found in most members of Coleoptera (beetles) and in most larvae of Lepidoptera (moths and butterflies); however, the rectal complexes found in these two insect Orders differ markedly in their anatomy and function (8–10), implying that the two structures likely evolved through convergent evolution. In *Tenebrio*, the water-conserving properties of the rectal complex are believed to rely on the active potassium chloride (KCl) transport by the PTs to generate a fluid of sufficiently high osmotic pressure to facilitate osmotically driven water removal from the feces (11, 12). In effect, the system establishes a standing gradient along the anterior–posterior axis of the complex to maximize fluid absorption in a manner analogous to the countercurrent arrangement of the vertebrate nephron in the renal medulla (13). In this way, *Tenebrio* (and its relatives) is able to extract and recycle almost all water from the rectal lumen to produce powder-dry excreta (7, 14). Remarkably, the system can even be used as a physiological mechanism for water uptake by enabling absorption of water vapor directly from the moist air (5–7, 14, 15). It has been suggested that the accumulation of KCl in the PTs is mediated by a small population of secondary cells known as “leptophragmata,” as they are the only cells that interrupt the perinephric membrane to enable hemolymph-to-tubule movement of KCl (6, 7). However, in spite of having been intensely studied for almost a century, the cellular and molecular architecture underpinning the water-extracting functions of this extraordinary organ remains largely unknown.

Cataloging the relative strength and specificity of gene expression across different tissues and life stages of an organism can provide valuable insights into most biological functions. Transcriptomic atlases have therefore become powerful tools in the functional genomics arsenal by enabling the annotation of physiological mechanisms and developmental processes on a gene-by-gene basis (16–20). However, despite their obvious benefits to their respective communities, the number of such transcriptomic atlases is limited. In the field of insect functional genomics, the red flour beetle *Tribolium castaneum* (a closely related species of *Tenebrio*) has emerged as a powerful model system because of its rapidly expanding transgenic toolkit (21–24) and its amenability to large-scale mutagenic studies (25, 26). The construction of an authoritative expression atlas for *Tribolium* would thus not only complement with existing postgenomic resources, but also help broaden the scope for functional analysis of one of the most economically (contain many devastating crop pests) and ecologically (largest group of insects) important animal groups on Earth, the beetles.

Here, we report the development of BeetleAtlas, a transcriptomic atlas covering distinct ontogenetic and tissue-specific expression profiles of *Tribolium*, and demonstrate the utility of this resource by identifying a cation/H^+^ antiporter (NHA1) that is essential to the water-extracting properties of the *Tribolium* rectal complex. Using a combination of bioinformatics, genetics, imaging, electrophysiology, and organ assays, we show that NHA1 localizes exclusively to the specialized leptophragmata cells in the PTs where it acts as an electroneutral cation/H^+^ antiporter. Genetic depletion of *Nha1* dramatically increases excretory water loss and impairs whole-animal survival during desiccation stress, suggesting that NHA1 activity is essential for maintaining systemic water balance. Finally, we show that NHA1 expression and leptophragmata maturation is regulated by a transcription factor called Tiptop, which is part of a conserved gene regulatory network that is central to the function of the rectal complex in tenebrionid beetles. Taken together, our work provides insights into the molecular architecture underlying the function of one of the most powerful water-extracting mechanisms in biology, the tenebrionid rectal complex.

## Results

### BeetleAtlas: A Transcriptional Atlas of *Tribolium* Tissues and Life Stages.

**Fig. 1.**
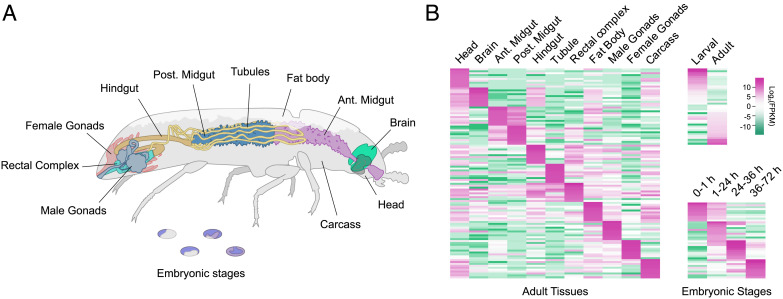
BeetleAtlas is a comprehensive transcriptomic atlas of gene expression. (*A*) Adult *Tribolium* anatomy highlighting the tissues selected for microdissection and bulk RNA sequencing. (*B*) Clustered heat maps of gene expression (log_2_ transformed values of fragments per kilobase of transcript per million, FPKM) for select genes that are enriched in a certain tissue or life stage in *Tribolium*. The same genes are depicted across all samples demonstrating distinct transcriptional signatures for each tissue or ontogenetic stage.

A large fraction of any animal genome is differentially expressed in distinct cell types, tissues, and life stages. Therefore, it is vital to consider spatiotemporal changes in gene expression in order to understand cell functions. To gain a comprehensive view of the transcriptional landscapes of individual tissues and life stages of *Tribolium*, we have created BeetleAtlas (BeetleAtlas.org). This is a transcriptional atlas of gene expression based on RNA sequencing (RNAseq) that covers several embryonic stages as well as major larval and adult tissues. Specifically, it catalogs transcriptomic datasets covering 11 distinct adult tissues (head, brain, anterior midgut, posterior midgut, hindgut, Malpighian tubules, rectal complex, fat body, female/male gonads, and carcass) dissected from 7-d-old San Bernardino adult beetles; nine larval tissues (head, brain, anterior midgut, posterior midgut, hindgut, Malpighian tubules, rectal complex, fat body, and carcass) dissected from L6 larvae; and four distinct (0 to 1, 1 to 24, 24 to 36, and 36 to 72 h post egg lay) stages of embryonic development (Fig. 1*A*). Each sample was prepared according to standardized protocol and sequenced on the same sequencing platform in a minimum of three biological replicates and compared to matched whole-animal samples. Together, BeetleAtlas thus allows systematic comparisons of gene expression across all major tissues and ontogenetic stages of more than 16,500 genes (with over 18,500 transcripts) encoded by the *Tribolium* genome (27).

An implicit requirement of such a facility is the robustness of the data in the underlying database. To formally assess the quality of our data, we identified the most highly expressed genes in each sample and generated a clustered heat map to visualize the divergence of transcriptomes between the different ontogenetic stages and larval and adult tissues (Fig. 1*B*). These data show a clear discrimination of the transcriptional signatures, not only between different life stages, but also between physically adjacent tissues within the same stage, such as the larval midgut and tubules or the adult rectal complex and gonads. Furthermore, we performed manual searches for genes that show a strong enrichment in a particular tissue and performed independent validation of gene expression by qRT-PCR. These results further confirm and validate the spatial expression profiles reported by BeetleAtlas across the selected genes (*SI Appendix*, Table S1). BeetleAtlas is thus a powerful tool for the *Tribolium* and insect communities, providing a critical first step by mapping gene activity to particular stages or tissues and by offering valuable information on the function of each gene product by integrating complementary large-scale RNA interference (RNAi) screening data (28).

### BeetleAtlas Identifies Candidate Genes Involved in Rectal Complex Function.

**Fig. 2.**
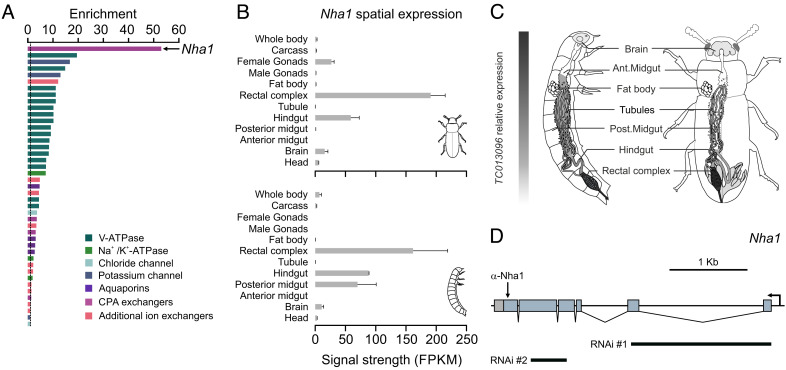
Mining BeetleAtlas to identify molecules involved in rectal complex function. (*A*) Fold increase (enrichment) in gene expression of membrane channels and transporters in the rectal complex relative to the whole animal (dashed line) according to BeetleAtlas. A gene encoding a putative Nha1-like protein is most highly enriched in the rectal complex. (*B*) Spatial expression analysis of *Nha1* shows that it is almost exclusively expressed in the hindgut and rectal complex of both larval and adult *Tribolium*. (*C*) Heat map of *Nha1* expression superimposed on larval and adult anatomy. (*D*) Predicted exon map of *Nha1* marking the epitope targeted for antibody generation and regions selected for dsRNA synthesis. Of the two fragments tested, fragment #2 produced the strongest knockdown and was therefore used in the remaining part of the study.

To demonstrate the utility of BeetleAtlas in providing transcriptional insights into tissue function, we aimed to characterize the molecular mechanisms underpinning the function of the rectal complex (6). Using the “Gene” lookup section of BeetleAtlas, we performed manual searches of all genes identified as core components of the insect “epitheliome” (29) and looked for transcript enrichment of the *Tribolium* orthologs relative to that of the whole animal. In addition, we used the “Tissue” search function to perform an unbiased search for candidate transporter genes that are most highly enriched in the rectal complex. Adopting parallel hypothesis-driven (candidate genes) and hypothesis-free (gene enrichment) approaches, we identified more than 30 putative genes that are expressed at very high levels in this multiorgan system compared to the organismal average, and further revealed that most of these genes belong to a core set that is coexpressed in other insect transport epithelia (29, 30). From this candidate list, *TC013096,* a gene predicted to encode a cation/H^+^-antiporter (*Tcas-Nha1,* hereafter just *Nha1*), shows the highest enrichment in the rectal complex (Fig. 2*A*). Indeed, mapping the tissue-specific expression pattern of *Nha1* indicates that this gene is predominantly expressed in the rectal complex of both larval and adult *Tribolium* (Fig. 2 *B*–*D*); an expression profile that we validated by qRT-PCR (*SI Appendix*, Table S1). Together, the transcriptional signature of *Nha1* suggests that this transporter is likely to play a key role in the function of the *Tribolium* rectal complex.

### NHA1 Localizes to a Specialized Cell Type in the Perirectal Tubules of the Rectal Complex.

**Fig. 3.**
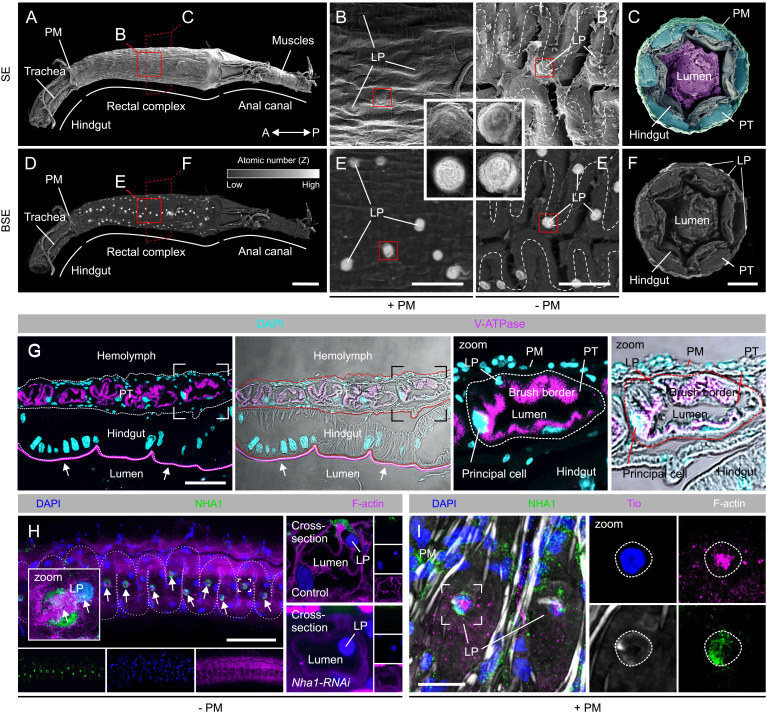
NHA1 localizes to specialized leptophragmata cells in the PTs of the rectal complex. (*A*–*C*) Scanning electron microscopy (SEM) images showing the gross morphology of the rectal complex. A subpopulation of cells is arranged along the perirectal tubules (PTs) as a series of dilations that extend radially toward the perinephric membrane (PM), suggesting that they are leptophragmata (LP). (*D*–*F*) Back-scattered electron (BSE) detection reveal that these dilations contain elements of high atomic number (*Z*) following AgNO_3_ application, confirming that these dilations are LP. (*G*) Maximum projection confocal microscopy images of paraffin sections of the rectal complex demonstrate that the V-ATPase localizes predominantly to the apical brush border of principal cells in the PTs (scale bar, 40 µm.) Note the cuticle of the rectal epithelium shows strong autofluorescence (arrows). (*H*) Maximum projection confocal microscopy images demonstrate that NHA1 is expressed in this subpopulation of LP cells along the PTs (small arrows). Zoom: note the small nucleus of the LP and the characteristic central band of F-actin spanning the apical surface of the cell (small arrows). Cross-section of PT: Subcellular localization of NHA1 demonstrates exclusive expression in the small-nucleated LP cells. Rectal complex dissected from animals injected with dsRNA targeting the *Nha1* gene (*Nha1-RNAi*) shows a dramatic reduction in immunoreactivity confirming the specificity of the antibody. (scale bar, 100 µm.) (*I*) LP identity is further confirmed by coexpression of the Tiptop (Tio) transcription factor, which is a marker of the LPs [as well as secondary cells in the “free” part of the tubule (34)].

To gain insight into the physiological roles of NHA1, we next examined the structure and spatial organization of the *Tribolium* rectal complex using scanning electron microscopy. Like the rectal complex from the closely related mealworm *Tenebrio* (6, 7, 31), the *Tribolium* rectal complex is organized according to the cryptonephridial condition. This condition is characterized by the distal ends of the MTs being closely applied to the rectal epithelium in a sinuous pattern and enclosed within a compartment, the perinephric space that is separated from the body cavity by an impermeable perinephric membrane (Fig. 3 *A*–*C*). Notably, the distal regions of the MTs—the so-called perinephric tubules (PTs)—are conspicuously different from the “free” part of the tubules as they are characterized by small dilations over which the perinephric membrane is extremely thin and in direct contact with the dilations (“boursourflors”) under which specialized small-nucleated cells known as leptophragmata are found (Fig. 3 *B* and *B*’). The anatomical position of these cells is intriguing as previous studies in *Tenebrio* had demonstrated that the function of the rectal complex is based on the accumulation of high concentrations of KCl in the PT lumen mediated by active hemolymph-to-tubule transport to facilitate osmotically driven water removal from the feces (6, 7, 12). Such a model is supported by the high expression of genes encoding the different subunits of the plasma membrane Vacuolar H^+^ ATPase (V-ATPase) (Fig. 2*A* and *SI Appendix*, Fig. S1*C*)—a plasma membrane transporter critical to energizing insect epithelia (32)—and by the exclusive localization of the protein to the apical brush border of the PTs in the *Tribolium* rectal complex (Fig. 3*G*). As the perinephric membrane is highly impermeable except for the “blister-like” windows under which leptophragmata sit (33), the anatomical data strongly suggest that these small cells are the only sites of exchange. Consistent with this notion, backscattered electron detection—a method that carries information on differences in atomic number (*Z*) of the sample—on rectal complexes preincubated in silver nitrate (33) reveals that silver staining is exclusively found at sites corresponding to the anatomical position of the leptophragmata (Fig. 3 *A*–*F*), implying that hemolymph-to-tubule transport is exclusively mediated by these cells. Strikingly, NHA1 protein was shown to localize entirely to a small-nucleated cell type resembling the leptophragmata of the PTs (Fig. 3*H*) using our custom-raised NHA1-specific antibody. The specificity of the antibody was verified by lack of immunoreactivity in *Nha1*-depleted animals (*SI Appendix*, Fig. S1 *A* and *B*). Indeed, the expression of NHA1 in leptophragmata was confirmed by double staining with Tiptop (Tio) (Fig. 3*I*), a transcription factor that marks leptophragmata and is involved in the differentiation of secondary cells in *Tribolium* and other insects (34–36). This finding suggests that the leptophragmata and the secondary cells of the free tubules are related. Taken together, our data indicate that the anatomical features of the *Tribolium* rectal complex are largely identical to those described previously for *Tenebrio* (6, 7), and that NHA1 is exclusively localized to the specialized leptophragmata cells in the PTs of the rectal complex.

### NHA1 Acts as an Electroneutral Cation/H^+^ Antiporter.

**Fig. 4.**
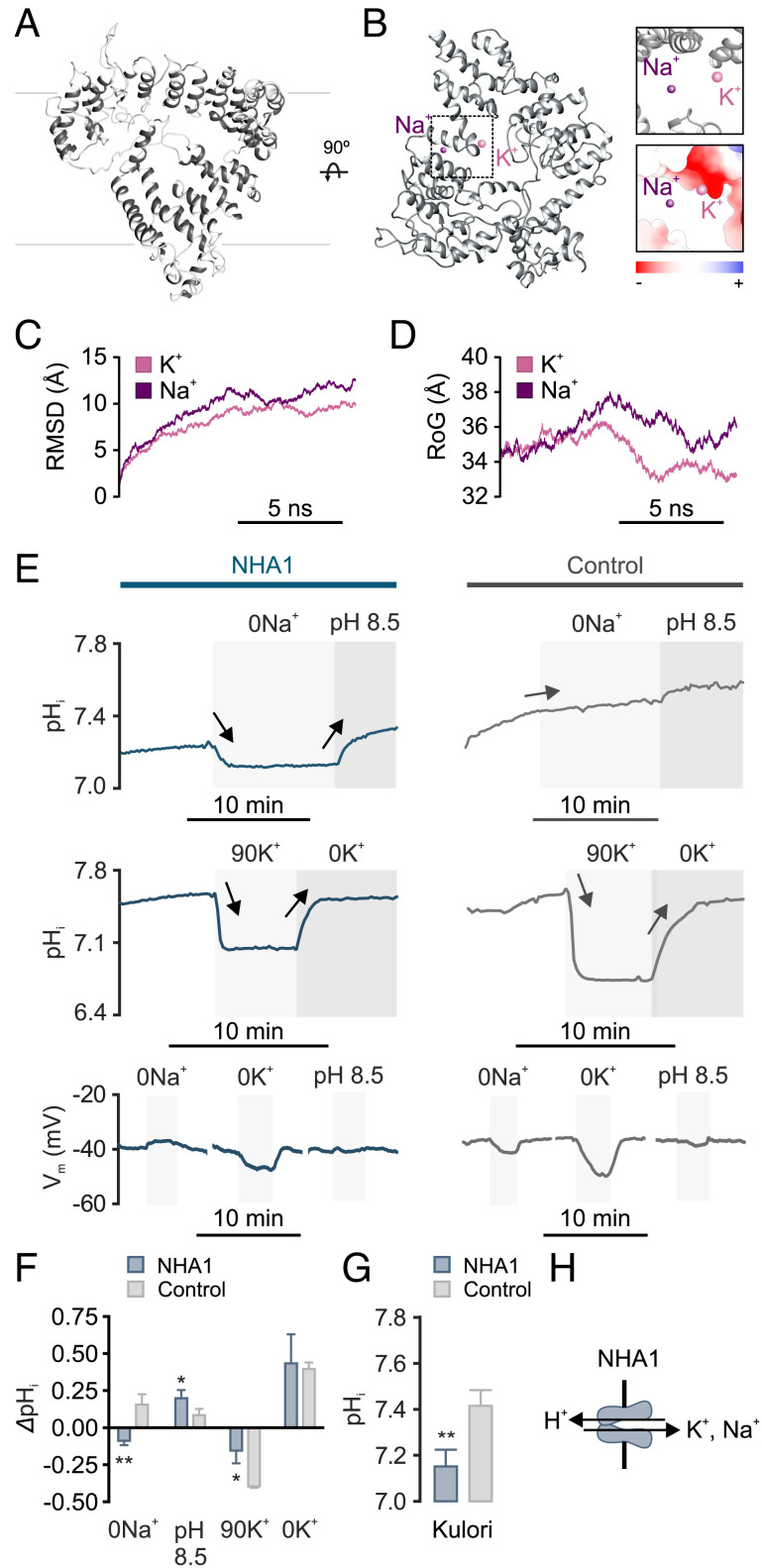
NHA1 acts as a K^+^(Na^+^)/H^+^ exchanger. (*A*) Predicted three-dimensional ribbon diagram of the NHA1 transporter embedded in the plasma membrane (*B*) *Top* view of the NHA1 protein highlighting K^+^ (pink) and Na^+^ (purple) binding; K^+^ is predicted to bind closer to negatively charged transport funnel compared to Na^+^ (*Insets*). (*C*) Root mean square deviation (RMSD) plot of NHA1 backbone (C-α) atoms in the presence of K^+^ (pink) and Na^+^ (purple) as a function of time. (*D*) Radius of gyration (RoG) plot of NHA1 compactness in the presence of K^+^ (pink) and Na^+^ (purple) as a function of time. (*E*) Representative traces of pH_i_ and V_m_ of control (dark gray) or *Nha1* (blue)-injected *X. laevis* oocytes in response to changes in bath pH, [Na^+^] and [K^+^]. The gray boxes mark replacement of the control Kulori’s solution (90 mM NaCl, 4 mM KCl, 1 mM MgCl_2_, 1 mM CaCl_2_, 5 mM HEPES, pH 7.4) with either (0Na^+^) Kulori’s solution where NaCl was substituted by choline-Cl (pH 8.5); where pH was adjusted to 8.5 (90K^+^); where the potassium concentration was increased or (0K^+^); where KCl was substituted by choline-Cl. Significant changes in pH_i_ relative to the previous solution are indicated by arrows. (*F*) Quantitative comparison of changes in pH_i_ of oocytes in response to different challenges (Student’s *t* test; n = 5 to 10, *P < 0.05, **P < 0.01). (*G*) Resting levels of pH_i_ of oocytes from NHA1 (blue) and water-injected controls (dark gray) after incubation in Kulori’s solution pH 7.4 (Student’s *t* test; n = 5 to 10, **P < 0.050.01). (*H*) Schematic representation of ion transport mediated by NHA1.

The NHA transporters belong to the Cation/Proton Antiporter-2 (CPA2) subfamily of proteins, which are known to participate in a broad range of transport mechanisms across all kingdoms of life (37). Classically, the NHAs are secondary active transporters that exchange sodium ions against protons, yet several CPA2 members have been shown to move different types of substrates (38, 39), suggesting that their molecular function cannot be inferred from structural similarity. To gain insight into the role of NHA1 in rectal complex physiology, we first performed sequence analysis and computational modeling of the protein. Surprisingly, we found only one *Nha* gene (*Nha1*) encoded by the *Tribolium* genome, which contrasts with other insect genomes in which two paralogs (*Nha1* and *Nha2*) are present (37, 38, 40). Interestingly, a BLASTp search against *Drosophila* proteins reveals that *Tribolium* NHA1 is more closely related to *Drosophila* NHA1 (53% sequence identity) than to NHA2 (35% sequence identity). Next, we performed three-dimensional structure predictions using I-TASSER (41) followed by Ramachandran plot and molecular dynamics simulations (42) to identify the putative transmembrane domains and globular structure of the protein. Consistent with the structural hallmarks of CPA2 members, the structural architecture of *Tribolium* NHA1 is defined by 12 transmembrane helices (both the N- and C-terminal tails are located in the cytoplasm) that collectively form a negatively charged transport funnel containing the putative ion binding and translocation domains (Fig. 4 *A* and *B* and *SI Appendix*, Fig. S2 *A–C* and Movie S1). These findings led us to perform molecular dynamics simulations in the presence of cation substrates (K^+^ or Na^+^) to help resolve the ion binding and cation selectivity of NHA1. After an initial phase of equilibrium, we observed that the structural stability of the protein was predicted to be higher (i.e., lower radius of gyration and root mean square deviation) in the presence of bound K^+^ relative to bound Na^+^ (Fig. 4 *C* and *D*), implying that K^+^ binding is favored over Na^+^ by the *Tribolium* NHA1 protein.

We next sought to experimentally validate our molecular dynamics simulations by cloning and heterologously expressing *Tribolium* NHA1 in *Xenopus laevis* oocytes to allow electrophysiological characterization of the protein, as described in ref. 43. Monitoring intracellular pH (pH_i_) in NHA1 oocytes while sequentially manipulating extracellular [Na^+^], [K^+^], and pH in the superfusing solution revealed that the oocytes rapidly responded to these changes, whereas similar responses were not observed in control oocytes injected with water (Fig. 4 *E* and *F*). These data suggest that *Tribolium* NHA1 is capable of recognizing both Na^+^ and K^+^ as substrates with either cation being exchanged for a H^+^. However, the significantly lower pH_i_ of resting NHA1 oocytes relative to water-injected controls (Fig. 4*G*) supports our in silico analysis predicting that K^+^ is favored over Na^+^ (Fig. 4 *C* and *D*). The membrane potential (V_m_) of NHA1 oocytes was insensitive to extracellular pH, implying that NHA1 transport is not electrogenic (Fig. 4*E* and *SI Appendix*, Fig. S3). Taken together, our results show that in *Tribolium*, NHA1 is functionally coupled to the H^+^ gradient to mediate electroneutral exchange of K^+^ (Na^+^) for H^+^ (Fig. 4*H*) in a manner similar to that observed for fungal KHA members (39, 44, 45).

### Internal Water Abundance Modulates NHA1 Activity in Leptophragmata Cells.

**Fig. 5.**
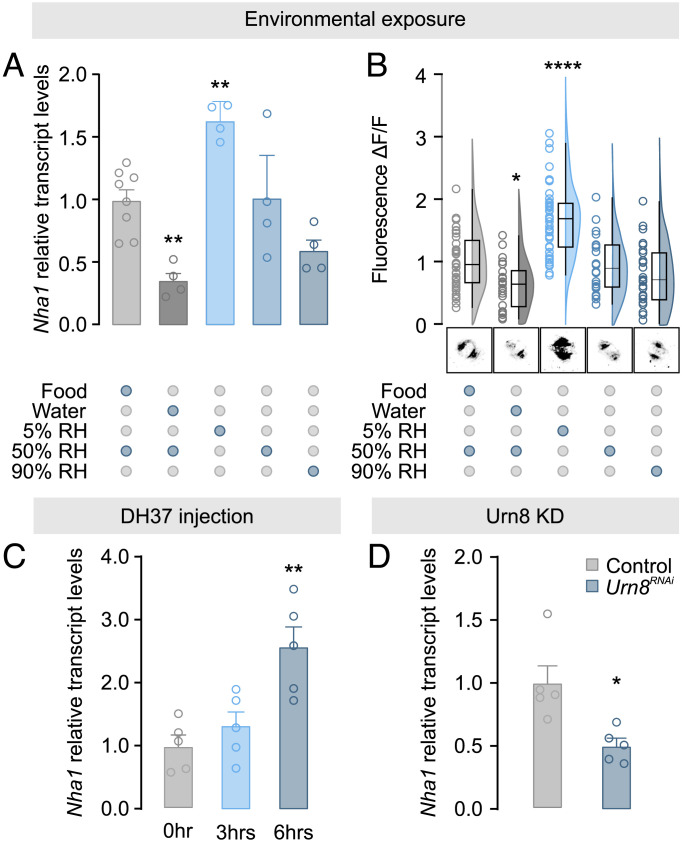
Environmental cues modulating rectal complex *Nha1* expression. (*A*) *Nha1* transcript levels in the rectal complex (*n* = 4 to 8) and (*B*) Raincloud plot of anti-NHA1 immunofluorescence (*n* = 23 to 45) in leptophragmata from animals exposed to different environmental conditions. Representative images of NHA1 immunofluorescence levels following exposure to each condition are shown below. Significant differences indicate pair-wise comparisons between control (food, RH 50%) and a given experimental group (one-way analysis of variance (ANOVA), *P < 0.05, ****P < 0.0001). (*C*) *Nha1* transcript levels in the rectal complex (*n* = 5) from animals injected with DH37 (one-way ANOVA, **P < 0.01) or (*D*) from beetles injected with dsRNA targeting the *Urn8* gene (unpaired Student’s *t* test, *P < 0.05).

The potent activation of the rectal complex observed in *Tenebrio* larvae deprived of water (6) implies that the transport machinery of the complex is regulated in response to changes in hemolymph osmotic pressure. We therefore asked whether NHA1 activity is altered in animals exposed to conditions known to affect internal water abundance (34, 46). Quantifying NHA1 expression in the rectal complex revealed that both transcript and protein levels were consistently decreased in animals exposed to conditions that promote fluid retention (water, relative humidity, RH 90%), and significantly increased in beetles exposed to severe desiccation (RH 5%), compared to control animals (Fig. 5 *A* and *B*). Furthermore, artificial activation (DH37 injection) and genetic deactivation (*Urn8* knockdown) of a potent diuretic pathway known to regulate organismal water levels (34) induced a robust increase and a significant decrease in *Nha1* expression, respectively (Fig. 5 *C* and *D*). These observations suggest that NHA1 abundance is regulated in response to internal changes in hemolymph concentration as part of homeostatic mechanism that modulates the reabsorptive capacity of the rectal complex to maintain ion and water balance. Such a role is consistent with the observation that the concentration of the perirectal fluid in animals exposed to desiccation increases disproportionately relative to the hemolymph, presumably to allow more effective reabsorption of water from the rectal lumen (6).

### Silencing *Nha1* Expression Increases Sensitivity to Desiccation by Inducing Excessive Water Loss.

**Fig. 6.**
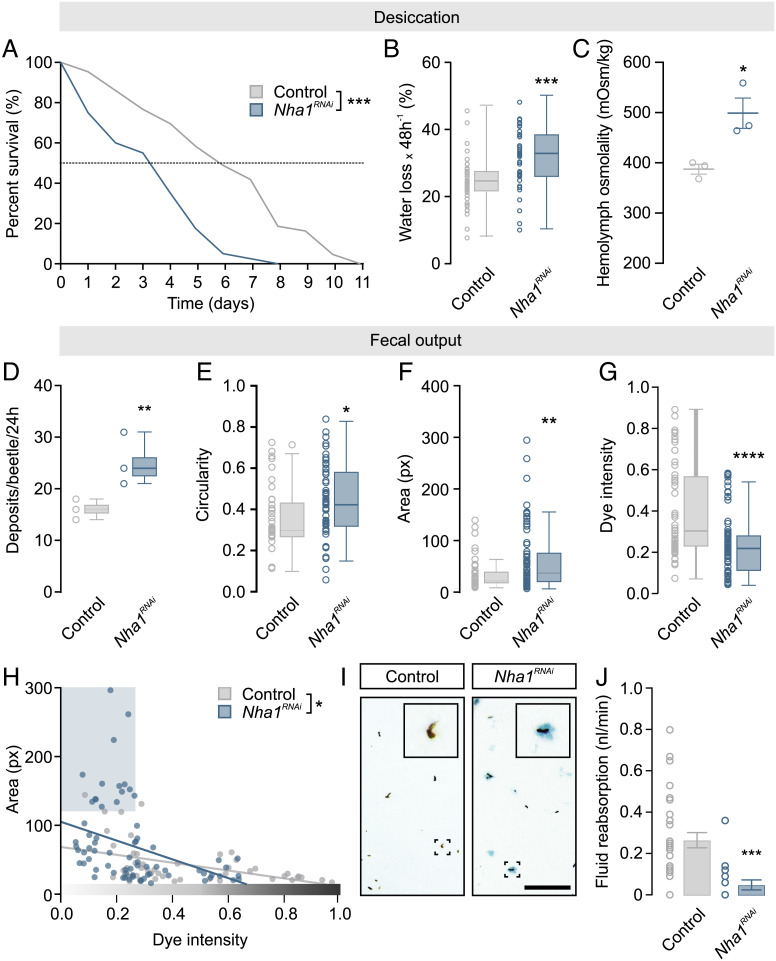
Systemic water balance depends on NHA1 activity. (*A*) Kaplan–Meyer survival function of adult control (dsRNA targeting *beta-lactamase, amp^R^*) and *Nha1*-silenced animals. Adult-specific knockdown of *Nha1* reduces organismal survival when exposed to low humidity conditions compared to control (RH 5%, log-rank test, *n* = 41 to 43). (*B*) Gravimetric analysis of control and *Nha1*-silenced animals. Desiccation-induced water loss is significantly increased in *Nha1* knockdown animals relative to control (unpaired Student’s *t* test, *n* = 57, ***P < 0.001). (*C*) Hemolymph osmotic pressure of control and *Nha1*-depleted beetles. Hemolymph osmolality is significantly increased in *Nha1* knockdown animals relative to controls (unpaired Student’s *t* test, *n* = 3, ***P < 0.05). (*D*) Defecation rate of *Nha1*-depleted animals is significantly increased relative to controls (unpaired Student’s *t* test, ***n = 3 groups with 10 animals per group, **P < 0.05) and (*E*) the deposits are more circular (*P > 0.05), (*F*) larger (**P < 0.01), and (*G*) with a reduced dye intensity (****P < 0.001). (*D–G*) Statistical differences were tested using unpaired Student’s *t* test. (*H*) The size of the deposits is inversely correlated with dye intensity in excreta from both *Nha1*-silenced (blue trend line) and control animals (gray trend line) yet the slopes of the trend lines are significantly different (*P < 0.05). Note the subpopulation of large, less intense deposits (blue shaded box), which is produced almost exclusively by *Nha1* knockdown beetles. (*I*) Representative images of excreta produced by control and *Nha1* knockdown animals. Deposits produced by *Nha1* knockdown animals were often associated with excess fluid, as evidenced by the dye-labeled fluid surrounding the deposit, which was never observed in control animals (scale bar, 1 mm.) (*J*) Ex vivo preparations of *Nha1* knockdown beetles show a significantly lower rectal complex-mediated fluid reabsorption rate relative to control (unpaired Student’s *t* test, *n* = 17 to 33, ***P < 0.001).

We next sought to explore the functional significance of NHA1 in maintaining systemic water balance in vivo by selectively down-regulating *Nha1* expression using RNAi. Given that the rate of water loss is a crucial factor in determining the tolerance to desiccation in *Tribolium* and other insects (34, 47, 48), we hypothesize that *Nha1* depletion might lead to increased excretory fluid loss and thus impair the ability of *Tribolium* to survive dry conditions. Consistent with this hypothesis, *Nha1*-silenced animals showed an increased sensitivity to desiccation relative to control-injected animals, with a median survival of 3.3 d as compared to 5.8 d (Fig. 6*A*); RNAi efficacy was verified by qRT-PCR and immunocytochemistry showing >95% knockdown and a complete loss of detectable NHA1 expression (*SI Appendix*, Fig. S1 *A* and *B*). This reduction in median survival is likely explained by an impaired ability to conserve water, since *Nha1* knockdown beetles consistently showed an increased rate of organismal water loss and a concomitant increase in hemolymph osmotic pressure (Fig. 6 *B* and *C*). These effects were retained, albeit to a diminished extent, in animals exposed to high humidity implying that NHA1 function is essential to maintain systemic water balance across a wide range of conditions of fluid stress (*SI Appendix*, Fig. S4 *A–C*). To test if the observed sensitivity to desiccation in *Nha1*-silenced beetles can be explained by increased excretory water loss, we examined the fecal output profiles of control and knockdown animals using an established in vivo excretion assay based on a dye-laced food source (34, 49, 50). These results showed that *Nha1* depletion resulted in increased defecation rate and excretory fluid loss as revealed by the production of more abundant, circular, larger, and less concentrated excreta as compared to control-injected animals (Fig. 6 *D*–*H*). Indeed, the deposits produced by *Nha1*-knockdown animals were often visibly associated with a striking increase in excess fluid as manifest by a “halo” surrounding the excreta, which was never observed in control-injected beetles (Fig. 6*I*). To test whether this increased excretory water loss was causally linked to defects in the reabsorptive capacity of the rectal complex, we adapted and optimized an ex vivo method based on isolating the entire alimentary canal under paraffin oil to quantify fluid reabsorption by the system (51); the preparations remained viable for several hours (*SI Appendix*, Fig. S5). These experiments revealed that *Nha1* knockdown almost completely abolished fluid reabsorption by the rectal complex (Fig. 6*J*), thus demonstrating that this is, at least partly, the site responsible for the observed reduction in fluid retention. Taken together, our data suggest that loss of NHA1 function in leptophragmata cells dramatically impairs water reabsorption by the rectal complex, which affects systemic water balance and reduces the ability of adult beetles to survive desiccating conditions.

### Tiptop-Induced NHA1 Expression Underlies Water Vapor Absorption by the Rectal Complex.

**Fig. 7.**
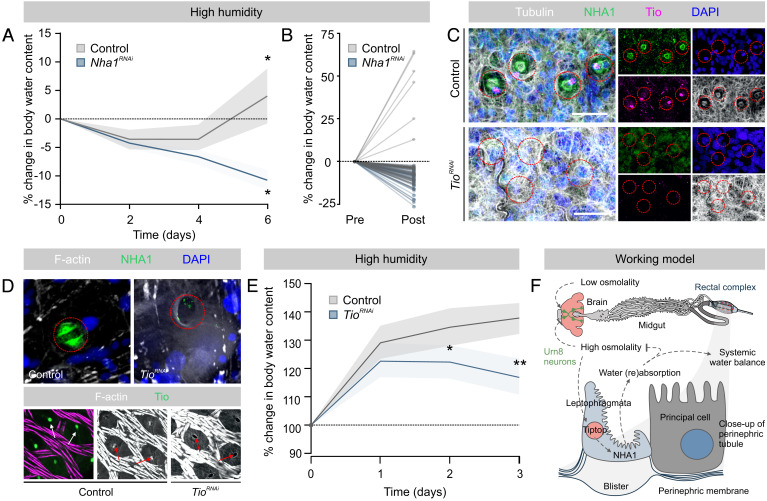
Tiptop controls NHA1 expression and leptophragmata differentiation. (*A*) Changes in body water in control and *Nha1*-silenced animals exposed to RH 90% as a function of time. *Nha1*-depleted animals consistently lose water over a 6-d period, while controls retain, or even increase, organismal water levels relative to their initial water levels (paired Student’s *t* test, *n* = 40 to 49, *P < 0.05). (*B*) Before–after plot of individual measurement graphed in *A* at day 0 (pre) and day 6 (post). (*C*) Immunolocalization of NHA1 and Tio in control and *Tio*-silenced animals. *Tio* knockdown results in almost complete loss of NHA1 expression as well as in morphological defects in the leptophragmata. (*D*) *Tio* depletion similarly abolishes NHA1 expression and causes defects in leptophragmata cytoarchitecture, such as a complete loss of the central band of F-acting spanning the LP (red arrows in *Bottom* panels), which (*E*) significantly impairs water vapor absorption compared to controls in larvae of *Tenebrio* (unpaired Student’s *t* test, *n* = 3 to 7, *P < 0.05; **P < 0.01). (*F*) Model for the systemic control of water balance in tenebrionid beetles.

The powerful water-extracting properties of the rectal complex are not only related to the reabsorption of water from the feces, but can also be coupled to the remarkable ability to absorb water vapor directly from moist air (5–7, 14). Given that this mechanism depends critically on generating sufficiently low water activities in the rectal lumen to allow condensation of water from the atmosphere (6), we hypothesized that *Nha1*-knockdown would impair the ability to perform water vapor absorption. As predicted, we found that *Nha1*-deficient animals consistently lost body water when deprived of food at high humidity, whereas control animals retained, or even gradually increased, body water during identical exposures (Fig. 7 *A* and *B*). These data confirm that *Tribolium* is able to absorb water at high relative humidities in a manner similar to that observed for *Tenebrio* (7, 14, 31)—albeit with a reduced capacity—and that this process depends critically on NHA1.

We next explored the gene regulatory networks that govern *Nha1* expression and leptophragmata physiology. The finding that Tio—a transcription factor involved in secondary cell differentiation in many insects (34–36)—is coexpressed with NHA1 in the leptophragmata of the PTs (Fig. 3*I*), suggests that Tio controls *Nha1* expression in leptophragmata. Accordingly, we selectively down-regulated *Tcas*-*Tio* (*TC012322*; hereafter just *Tio*) expression during early development and subsequently probed for NHA1 expression in the rectal complex of adult beetles. The results revealed that *Tio* knockdown caused a complete loss of NHA1 expression, and further induced overt defects in the cytoarchitecture of the leptophragmata as evidenced by the failed formation of blister-like windows in the perinephric membrane (Fig. 7*C*). To test whether these defects affected whole-animal physiology, we silenced *Tio* expression in larvae of *Tenebrio* (the larger size makes them more amenable to gravimetric studies) and assessed their ability to absorb water vapor (6). As observed in *Tribolium*, knocking down *Tio* in *T. molitor* caused an almost complete loss of NHA1 expression as well as clear morphological changes, such as a loss of the central band of F-actin, to the leptophragmata of the rectal complex (Fig. 3*H* and Fig. 7*D*). Furthermore, silencing *Tio* expression consistently impaired their ability to absorb water as the body water contents of knockdown animals were significantly lower than those of mock-injected controls (Fig. 7*E*). Taken together, our study identifies Tio as a key regulator of NHA1 expression and leptophragmata differentiation, which is essential to the function of the rectal complex and to the maintenance of systemic water balance in *Tribolium* (Fig. 7*F*).

## Discussion

### The Transport Functions of the Specialized Leptophragmata Underpin the Water-Conserving Functions of the Rectal Complex.

Classically, absorption of water by Tenebrionid beetles when exposed to subsaturated air was believed to occur across the cuticle, but it has since emerged that this process is entirely performed by the modified rectal complex (6, 7, 15, 31). In this work, we demonstrate that NHA1 in the specialized leptophragmata cells of the PTs is essential for the water-conserving functions of the rectal complex as well as the homeostatic control of water balance in *Tribolium* (Fig. 7*F*). How might we explain these physiological effects? Our combined analysis of the NHA1 protein suggest that, while it is capable of handling both Na^+^ and K^+^ movement, the main transport function of NHA1 is likely to mediate K^+^/H^+^ exchange. If this transport modality is replicated in intact animals, these data strongly imply that NHA1 is functionally coupled to the active uptake of K^+^ by the complex, and thus to generating the osmotic forces necessary to facilitate water removal from the feces. Consistent with this idea, *Nha1* depletion results in impaired fluid reabsorption by the rectal complex, increased excretion, and reduced organismal water levels. Moreover, both NHA1 transcript and protein are up-regulated in response to cues related to internal water stress. However, a difficulty presents itself when trying to reconcile these observed physiological effects with the fine structure of the rectal complex. Our work and previous studies (6, 7, 12) suggest that the main route of active KCl transport into the system is through the specialized leptophragmata, but these cells do not possess the anatomical hallmarks of active ion-transporting cells. The leptophragmata lack basal infoldings, have a reduced brush border, and contain few mitochondria (31, 52). It would therefore seem unlikely that these cells are solely responsible for mediating the high KCl concentrations measured in the PT lumen (6). By contrast, the larger principal cells possess extensive basal infoldings, contain an extensive brush border full of mitochondria (7, 31), and are characterized by high expression of the plasma membrane V-ATPase (Fig. 3*G*). We therefore speculate that the H^+^ electrochemical gradient generated by the V-ATPase in the principal cells could drive the secondary active transport of K^+^ via NHA1 in the leptophragmata, with Cl^-^ following passively (7) to produce the necessary osmotic gradients. Such a model is supported by the recent observation that luminal-directed K^+^ secretion is mediated by the secondary cells of the “free” parts of the MTs in *Tribolium* (34), and is further analogous to that proposed for Na^+^ uptake by NHA2 in the secondary cells of MTs from the mosquito *Anopheles gambiae* (53). However, *Nha1* is not expressed in the “free” parts of the tubule in *Tribolium* (BeetleAtlas.org), and Urn8R—a hormone receptor diagnostic of the secondary cell identity—was not detectably expressed in the leptophragmata cells (34), implying that the PTs of the rectal complex operate via a two-cell type model that is distinct from that of the free tubules (34, 35). Mapping the exact routes and mechanisms with which water and ions flows through this multiorgan system remains an exciting prospect for the future. This could be addressed by single-cell RNAseq analysis of the rectal complex to obtain cell-specific insights into the molecular machinery that underlies the actions of the PT and rectal epithelia to help understand one of the most powerful water-conserving systems in biology.

### Exploring the Transcriptional Landscapes of *Tribolium*.

Transcriptomic atlases are powerful tools in molecular genetics as they offer a detailed spatio-temporal view of gene expression that provides valuable clues to each gene’s physiological function (16, 20, 54, 55). Here, we introduce BeetleAtlas; a transcriptomic resource that provides a comprehensive view of the genetic signatures that underpin the functions of distinct tissues or life stages in the genetic model organism *T. castaneum* (Fig. 1 *A* and *B* and *SI Appendix*, Table S1). Besides being a useful addition to the rapidly expanding toolbox for *Tribolium* research, BeetleAtlas will broaden the focus of the beetle community. *Tribolium* is often adopted for studies in evolutionary development; but by cataloguing gene expression across different life stages, BeetleAtlas can help inform a much wider range of biological questions. This is significant because, although systemic RNAi is robust at all stages of development (24, 56), this technique precludes tissue-specific gene interference, and so the tissue(s) that contribute to a given phenotype remain unknown. Our online resource can thus provide a useful filter with which to identify the major tissue(s) in which candidate genes are most abundantly expressed and therefore most conveniently studied. Such an approach might be particularly useful for the *post hoc* analysis of data derived from large-scale functional screens (25, 28, 57). To further encourage this utility, BeetleAtlas is fully linked to existing databases (iBeetleBase (28) and FlyAtlas (16)) by a common vocabulary, enabling access to complementary information (e.g., RNAi phenotype and *Drosophila* ortholog relations) that will promote its use and integration in future studies.

In addition to allowing a simple gene-by-gene lookup, there is also scope for meta-analyses of the underlying transcriptomic datasets. For example, rather than adopting a candidate gene approach based on already-known processes, it is possible to use a hypothesis-free method by asking: “Which genes are uniquely expressed in the adult brain, as compared to the larval brain?”. Such a methodology has the potential to generate unexpected research hypotheses independent of prior knowledge by allowing unbiased insights into the genes that underpin the functions of individual tissues or life stages (34, 58, 59). As well as offering a global analysis of gene expression, there is further information to be extracted by querying the underlying relational database. The GAL4/UAS binary expression system is introduced in *Tribolium* (60), yet most GAL4 drivers may not be entirely specific to particular life stages of individual tissues. Genes with tissue-specific or ontogenetically restricted roles can be easily identified using BeetleAtlas (e.g., *SI Appendix*, Table S1), and used to make GAL4 driver lines with tissue- or stage-specific activities. Conversely, genes with ubiquitous expression or with persistent roles throughout development can also be found. In sum, we predict that this online resource will have a major impact on the insect functional genomics community by providing an extensive catalog of gene expression across different tissues and life stages in *Tribolium*, which will promote both an evolutionary and an ontogenetic and tissue-centered view of gene function.

## Materials and Methods

### Animal Husbandry.

*Tribolium castaneum* (San Bernardino strain) stocks were maintained on organic whole-wheat flour supplemented with 5% (w/w) yeast powder (*Tribolium* medium) at 30 °C at a constant 50% relative humidity (RH) and 12:12 light–dark cycles as in ref. 61. For further details, **SI Appendix*, Materials and Methods*.

### Tissue Dissection and RNA Extraction.

Tissues were dissected and total RNA was extracted from embryos, nonsedated 6th instar larvae or 1-wk-old mature adults under a freshly prepared mixture of Schneider’s medium (Invitrogen, CA, US) and *Tribolium* saline (1:1, v/v) as described in **SI Appendix*, Materials and Methods*.

### RNA-seq Analyses and Database Construction.

Total RNA libraries were sequenced on a BGISEQ-500 using paired-end chemistry, and subsequent bioinformatic analyses were performed using the Tuxedo pipeline (62). These datasets were used to populate a database, TriboliumDB, that underlies a web application, BeetleAtlas, which is publicly available at www.BeetleAtlas.org. The web application employs a Java servlet to generate web pages and communicate with the TriboliumDB database, and separate smaller servlets for subsidiary functions. It contains a documentation section with full details and version dates. As a web application, BeetleAtlas thus allows nontechnical users to make a wide range of prepared queries to the underlying TriboliumDB. For this study, the BeetleAtlas web application was interrogated for gene orthologs of known ion channels and transporters (“Gene” lookup function) as well as for genes enriched in the rectal complex relative to the whole-animal signal (“Tissue” enrichment function), with all candidate genes prioritized according to enrichment. For further information, see **SI Appendix*, Materials and Methods*.

### Scanning Electron Microscopy (SEM).

SEM analysis of the rectal complex was performed according to a modified protocol described in ref. 58. In brief, rectal complexes were dissected under Schneider’s medium and briefly exposed to a AgNO_3_ solution (30 s) as described in ref. 33 before being fixed in 2.5% glutaraldehyde in 0.1M cacodylate buffer (pH 7.4) for 90 min as in ref. 49. The tissue was then desiccated and coated with platinum (70 s B12 nm thickness) and examined with a Zeiss Sigma variable-pressure scanning electron microscope (Carl Zeiss, Oberkochen, Germany) using secondary electron and back-scatter electron detection methods to sequentially visualize both the topology and element weight distribution (atomic number, *Z*) of the samples. For further information, see **SI Appendix*, Materials and Methods*.

### Antibody Generation and Immunolocalization of Target Proteins.

To generate anti-NHA1 and anti-VHA55 specific antibodies, we analyzed the amino acid (aa) sequence of the proteins to identify the optimal immunizing peptide region according to a previously described method (63). These antibodies were subsequently used for immunocytochemistry (58) to visualize immunofluorescence in different tissues. Where necessary, immunofluorescence levels were quantified using the FIJI software package from images acquired using identical microscope settings as described in ref. 34. For further information, see **SI Appendix*, Materials and Methods*.

### Computational Modeling and NHA1 Structure–Function Predictions.

The three-dimensional tertiary structure of NHA1 was predicted by using I-TASSER followed by Ramachandran Plot Assessment (RAMPAGE) to perform structural refinement of the model. Finally, we performed dynamic stability estimations of NHA1 in the presence of either Na^+^ or K^+^ ions, by submitting the protein for 10 ns molecular dynamics simulation using AMBER18 on the Computerome 2.0 high-performance computing cluster as described in **SI Appendix*, Materials and Methods.*

### Molecular Cloning of *Nha1*.

cDNA of *Nha1* was synthesized from total RNA extracted from adult *T. castaneum* rectal complexes using the High-Capacity cDNA Reverse Transcription Kit with RNase Inhibitor (ThermoFisher, MA, USA), and the coding region of the gene was amplified using Q5® Hot Start High-Fidelity 2× Master Mix (New England Biolabs, MA, USA) using *Nha1*-specific primers (*SI Appendix*, Table S2). The PCR products were subsequently cloned into pGEMHE *X. laevis* oocyte expression vector using In-Fusion® HD cloning kit (TaKaRa Bio Inc, Kusatsu, JP) and the final sequence validated (Eurofin, Luxemburg, LU).

### pH Measurements in *X. laevis* Oocytes.

*Tribolium Nha1* was cloned and heterologously expressed in *X. laevis* oocytes to characterize the molecular function of the protein as described in detail in **SI Appendix*, Materials and Methods*.

### Gene Expression Analysis.

Validation of RNAi-mediated gene knockdown (performed 3 d post dsRNA injection) and environmentally induced changes in gene expression (*SI Appendix*, Table S2) was assessed by quantitative real-time PCR (qPCR) as described in **SI Appendix*, Materials and Methods*.

### Production of dsRNA and RNAi-Mediated Knockdown.

dsRNA synthesis and knockdown of target gene expression by RNAi was carried out according to a protocol described in **SI Appendix*, Materials and Methods.* Gene-specific primers that were tagged with T7 promoter sequences at both the 3′ and 5′ ends are listed in *SI Appendix*, Table S2.

### Environmental Stress Exposure.

Beetles were subjected to different environmental stressors as described in **SI Appendix*, Materials and Methods.*

### Desiccation Tolerance.

Desiccation tolerance of control and knockdown animals exposed to different relative humidities was assessed as described in **SI Appendix*, Materials and Methods*.

### Hemolymph Collection and Quantification.

Hemolymph was collected according to a modified protocol (34, 49, 64) from animals exposed to different environmental stress exposures. See **SI Appendix*, Materials and Methods* for further information.

### Quantification of Water Content.

Gravimetric estimates of total body water were made by measuring wet and dry body weight of each animal after a given environmental exposure in control and *Nha1* knockdown animals; see **SI Appendix*, Materials and Methods* for further details.

### Defecation Behavior.

The effects of manipulating *Nha1* expression on in vivo whole-animal excretory behavior was performed as described in (34) with further details provided in **SI Appendix*, Materials and Methods.*

### Ex Vivo Fluid Reabsorption Assay.

The water reabsorption rate from the rectal complex ex vivo was assessed using a modified protocol (51) and as described in **SI Appendix*, Materials and Methods*.

### Water Vapor Absorption Assay.

The ability to extract water vapor directly from the atmosphere was quantified gravimetrically as described in (5). Further details are provided in **SI Appendix*, Materials and Methods.*

### Statistics.

Data analysis was performed for each experimental condition using relevant methods as described in **SI Appendix*, Materials and Methods.*

## Supplementary Material

Appendix 01 (PDF)

Movie S1. Three-dimensional tertiary structure of NHA1 as predicted by I-TASSER and subsequent Ramachandran plot analysis for structural refinement with putative Na^+^ and K^+^ binding sites as predicted by molecular dynamics simulation using the AMBER suite on the Computerome 2.0 high-performance computer cluster.

## Data Availability

All study data are included in the article and/or supporting information.
