# Supplementary material for: NHA1 is a cation/proton antiporter essential for the water-conserving functions of the rectal complex in Tribolium castaneum
Source: Proc Natl Acad Sci U S A. 2023 Mar 21;120(13):e2217084120. doi: 10.1073/pnas.2217084120 (PMC10068851; doi:10.1073/pnas.2217084120)
Supplement: Supplementary file 1 — Appendix 01 (PDF) [file pnas.2217084120.sapp.pdf]

PNAS

**Supporting Information for**

# NHA1 is a cation/proton antiporter essential for the water-conserving functions of the rectal complex in *Tribolium castaneum*


Muhammad Tayyib Naseem[1], Robin Beaven[2], Takashi Koyama[1], Sehrish Naz[1], Sheng-yuan Su[2], David P. Leader[3], Dan Klaerke[4], Kirstine Calloe[4], Barry Denholm[2], and Kenneth Veland Halberg[1,*]

[1] Department of Biology, University of Copenhagen, DK-2100 Copenhagen, Denmark
[2] Biomedical Sciences, University of Edinburgh, Edinburgh EH8 9AG, UK
[3] Institute of Molecular, Cell and Systems Biology, University of Glasgow, Glasgow G12 8QQ, UK
[4] Department of Veterinary and Animal Sciences, University of Copenhagen, DK-1870 Frederiksberg, Denmark

Corresponding author: Kenneth Veland Halberg
Email: kahalberg@bio.ku.dk


**This PDF file includes:**

SI Materials and Methods
Figures S1 to S5
Tables S1 to S2
Movie S1 legend
SI References

## SI Materials and Methods

**Animal Husbandry.** *Tribolium castaneum* (San Bernardino strain) stocks were maintained on organic whole-wheat flour supplemented with 5% (w/w) yeast extract powder (*Tribolium* medium) at 30°C at a constant 50% relative humidity (RH) and 12:12 light-dark cycles as in (1). *Tenebrio molitor* was cultured on organic outmeal supplemented with 1% yeast extract powder, 3% organic polenta powder and occasional carrot slices under identical environmental conditions.

**Tissue Dissection and RNA Extraction.** Tissues were dissected from non-sedated 6th instar larvae or 1-week-old mature adults under a freshly prepared mixture of Schneider's medium (Invitrogen, CA, US) and *Tribolium* saline (1:1, v/v). The *Tribolium* saline contained: NaCl 90 mmol $l^{-1}$, KCl 50 mmol $l^{-1}$, $MgCl_2$ 5 mmol $l^{-1}$, $CaCl_2$ 2 mmol $l^{-1}$, $NaHCO_3$ 6 mmol $l^{-1}$, $NaH_2PO_4$ 6 mmol $l^{-1}$, Glucose 50 mmol $l^{-1}$ and the pH was adjusted to 7.0. Dissected tissues were then transferred to 500 µl QIAzol (Qiagen, Hilden, DE) and stored at –80°C until sufficient tissue had been collected to allow extraction of a minimum of 100 ng RNA in total. Next, the samples were thawed and physically disrupted using a beadmill (1 min max speed) using a TissueLyser LT (Qiagen, Hilden, DE) and then extracted with phenol-chloroform including an extra chloroform step and several RNA washing steps. The RNA was then finally purified using a Qiagen RNeasy Plus mini kit according to the manufacturer's instructions. The optional DNase step, the optional drying of the column, and back-elution were all included. For each sample, the concentration of RNA was determined using a NanoDrop 1000 Spectrophotometer (ThermoFisher, MA, USA), and the quality of the RNA was determined using an Experion Pro260 (Bio-Rad, CA, USA) with Experion RNA HighSens Analysis Kit (BioRad, CA, USA), according to the manufacturer's instructions. Each tissue sample was prepared in biological triplicates.

**RNA-seq Analyses and Database construction.** Total RNA libraries were prepared for each sample according to a low-input protocol by BGI Genomics (Shenzhen, Guangdong, China), and sequenced on a BGISEQ-500 using paired-end chemistry (100 nt reads) with a sequencing depth of 6 Gb per sample (i.e. approaching 40x of the ~150 Mb *Tribolium castaneum* genome). The resulting fastQ.gz files were processed through the Tuxedo pipeline (2) using version 5.2 of the *T. castaneum* reference genome assembly (3), and the output used to populate a MySQL relational database, entitled TriboliumDB. The database also contains gene data from the Tcas 5.3 reference genome, gene ontology information from the Gene Ontology Consortium (www.geneontology.org), and *Drosophila melanogaster* symbol and name information from FlyBase (flybase.org).

The database, TriboliumDB, underlies a web application, BeetleAtlas, publicly available at www.BeetleAtlas.org. The web application employs a Java servlet to generate web pages and communicate with the TriboliumDB database, and separate smaller servlets for subsidiary functions. It contains a documentation ('Docs') section with full details and version dates. As a web application, BeetleAtlas thus allows non-technical users to make a wide range of prepared queries to the underlying relational TriboliumDB. This database is freely available for download, so that more sophisticated custom queries can be made, if required, by those with access to basic bioinformatics expertise. For this study, the BeetleAtlas web application was interrogated for gene orthologs of known ion channels and transporters ('Gene' lookup function) as well as for genes enriched in the rectal complex relative to the whole-animal signal ('Tissue' enrichment function), with all candidate genes prioritized according to enrichment.

**Scanning electron microscopy (SEM).** SEM analysis of the rectal complex was performed according to a modified protocol described in (4). In brief, rectal complexes were dissected under Schneider's medium and briefly exposed to a $AgNO_3$ solution (30 sec) as described in (5) before being fixed in 2.5% glutaraldehyde in 0.1M cacodylate buffer (pH 7.4) for 90 min as in (6). The tissue was then rinsed repeatedly in $ddH_2O$ before being dehydrated through a graded ethanol series, and desiccated using an Autosamdri-815 critical point dryer (Tousimis Research Corporation, Maryland, USA). The rectal complexes were then transferred to aluminum stubs, fractioned and coated with platinum (70 s B12 nm thickness) in a JEOL JFC-2300HR high-

resolution fine coater (Jeol, Tokyo, Japan) and examined with a Zeiss Sigma variable pressure scanning electron microscope (Carl Zeiss, Oberkochen, Germany) using secondary electron (SE) and back-scatter electron (BSE) detection methods to sequentially visualize both the topology and element weight distribution (atomic number, *Z*) of the samples.

**Antibody Generation and immunolocalization of target proteins.** To generate specific antibodies against proteins of interest, we analyzed the amino acid (aa) sequence of the proteins to identify the best immunizing peptide region according to a previously described method (7). For NHA1, this analysis resulted in the selection of a peptide corresponding to aa 547–562 (SMSTTVSQKDSPKGE) in the C-terminal region of the full-length parent protein, which was then submitted for a custom immunization protocol carried out by Genosphere Biotechnologies (Paris, France). Additionally, aa 491-496 (PAATLAEFYPRDSRH) of the VHA55 peptide was also selected for preparation of polyclonal antisera. Epitope specificity of the different antisera was established by comparing wild-type and RNAi animals by immunostaining.

Immunohistochemistry on paraffin sections was carried out as in (8). Briefly, rectal complexes were dissected and fixed in 4% paraformaldehyde in PBS for 30 min before being dehydrated in a graded series of ethanol (70%, 90%, 99%) followed by incubation in xylene for 20 min. Next the tissues were incubated in paraffin for 30 min (3x exchanges with fresh paraffin) and embedded in paraffin wax in a suitable mold and left to cool for 2 days. Then, semithin sections were cut on a Leica ultramicrotome EM UC6 (Leica Microsystems, Wetzlar, Germany) with glass knives and mounted on objective slides. Slides were then dewaxed in Histo-Clear (Scientific Laboratory Supplies, US) for 15 min, hydrated in a decreasing series of ethanol (99%, 90%, 70%), and finally washed in dd$H_2O$ for 30 sec. Slides were finally stored in PBS and immunostained using out anti-VHA55 antibody as described below.

Immunocytochemistry (ICC) was performed as in (4). In brief, rectal complexes were dissected and fixed as described above. Tissues were then washed four-six times in PBST (PBS + 0.1% Triton X-100), blocked with PBST containing 2 % normal goat serum (blockPBST; Sigma-Aldrich, MO, USA) for 1 h, and incubated in primary antibodies. Primary antibodies used were polyclonal rabbit anti-NHA1 (1:500), polyclonal mouse α-VHA55 (1:500) and polyclonal rat α-Tio (9) (1:500). The subcellular location of the endogenous proteins were visualized by applying Alexa Fluor 488/647 anti-rabbit, anti-mouse or anti-rat secondary antibodies (1:500; Sigma Aldrich, MO, USA) in combination with DAPI (1:1000) and Rhodamine-conjugated Phalloidin (1:500; Sigma Aldrich, MO, USA) in blockPBST overnight at 4°C. Following several washes, first in PBST and then in PBS, the different tissues were mounted on poly-L-lysine coated 35mm glass bottom dishes (MatTek Corporation, MA, USA) in Vectashield (Vector Laboratories Inc., CA, USA) and imaged on an inverted Zeiss LSM900 confocal microscope equipped with airy scan 2 technology (Zeiss, Oberkochen, Germany). Where necessary, immunofluorescence was quantified using the FIJI software package from images acquired using identical microscope settings as described in (6).

**Computational Modeling and NHA1 Structure-Function Predictions.** The 3-dimensional tertiary structure of NHA1 was predicted by using I-TASSER (10) and a best-fit model was selected based on its confidence score (C-score). Further, Ramachandran Plot Assessment (RAMPAGE) was utilized to calculate the torsional angles and side chain conformations of all amino acid residues contained in the NHA1 protein sequence to perform structural refinement of the model. Finally, we performed dynamic stability estimations of NHA1 in the presence of either $Na^+$ or $K^+$ ions, by submitting the protein for 10 ns molecular dynamics simulation using AMBER18 (11) on the Computerome 2.0 high-performance computing cluster. System preparation including minimization and equilibrium processe were performed as previously described (12). The CPPTRAJ module incorporated in AMBER suite was used for trajectory analysis according to their root mean square deviation (RMSD), radius of gyration (RoG), and

hydrogen bond calculations. During the entire simulation period, plots presented the comparative stability of modelled NHA1 in the presence of $Na^+$ or $K^+$ relative to each other.

**Molecular Cloning of *Nha1*.** cDNA of *Nha1* was synthesized from total RNA extracted from adult *T. castaneum* rectal complexes using the High-Capacity cDNA Reverse Transcription Kit with RNase Inhibitor (ThermoFisher, MA, USA), and the coding region of the gene was amplified using Q5® Hot Start High-Fidelity 2X Master Mix (New England Biolabs, MA, USA) using *Nha1*-specific primers (*SI Appendix* Table S2). The PCR products were subsequently cloned into pGEMHE *Xenopus laevis* oocyte expression vector using In-Fusion® HD cloning kit (TaKaRa Bio Inc, Kusatsu, JP) and the final sequence validated (Eurofin, Luxemburg, LU).

**pH Measurements in *Xenopus laevis* Oocytes.** cRNA was generated from *Nha1* in PGEMHE using mMessage mMachine (Ambion, Austin, TX, USA). *X. laevis* oocytes (Ecocyte Bioscience, Dortmund, Germany) were injected with 50 nl cRNA solution containing 25 ng *Nha1* cRNA per oocyte using a micro-injector (Nanoject, Drummond Broomall). The oocytes were incubated 4-7 days hours at 19˚C in Kulori's solution (90 mM NaCl, 4 mM KCl, 1 mM $MgCl_2$, 1 mM $CaCl_2$, 5 mM HEPES, pH 7.4) before making pH recordings. pH-electrodes were from borosilicate glass capillaries with filament (120F-3, WPI) using a vertical puller (Narishige Scientific Instrument Lab). The resistance of the pipettes was 3-4 MOhm when filled with 3 M KCl and submerged in Kulori´s solution. The pipettes were baked for 2 hr at 220 °C in a metal box. Dimethyldichlorosilane (Silanization Solution I, Sigma Aldrich) was added to the box though the hole in the lid and the pipettes were silanized at 220 °C for 2-4 hr. The tip of the silanized pipettes was filled with a $H^+$-selective ionophore cocktail (hydrogen ionophore I cocktail A, Sigma-Aldrich) by dipping the tip of the pipette into the solution. The electrodes were back-filled with a solution containing 40 mM $KH_2PO_4$, 23 mM NaOH and 150 mM NaCl, pH 7.5. Next, a pH electrode was mounted on the head stage of an EPC7 amplifier, and calibrated using 100 mM KCl with 10 mM MES/TRIS pH 5.5, pH 6.6 and pH 7.5 before each recording and after each recording. The range was typically 55-60 mV/pH. If the voltage had shifted more than 5 mV, the recording was discharged. *X. laevis* oocytes were placed in a recording chamber and superfused with a standard Kulori's solution and impaled with the pH electrode. After 10 min the standard Kulori's solution was replaced by 1) a sodium free Kulori's solution where NaCl was substituted by choline-Cl ($0Na^+$) for 10 min then 2) by Kulori's solution, pH adjusted to pH 8.5 (pH 8.5) for 10 min. A second round of experiments, the standard Kulori's solution was replaced by 2) a high potassium Kulori's solution containing 15 mM NaCl and 90 mM KCl, 1 mM $MgCl_2$, 1 mM $CaCl_2$ og 5 mM HEPES, pH 7.4 ($90K^+$) for approx. 5 min and then superfused by 4) the same solution with KCl substituted with cholin-Cl ($0K^+$). The effects of the different solutions on the membrane potential were tested and two electrode voltage clamp were done using an Oocyte Clamp OC-725B (Warner Instruments). The pipettes were filled with 3 M KCl solution and the reference electrodes were connected to the bath via agar bridges with 3 M KCl. The signal in the voltage recording electrode was subtracted from the signal in the pH electrode to calculate the pH potential based on the calibration curves.

**Gene expression analysis.** Validation of RNAi-mediated gene knockdown and environmentally induced changes in gene expression was assessed by quantitative Real-Time PCR (qPCR). Total RNA extraction was carried out three days after dsRNA injection unless otherwise stated and cDNA synthesis were carried out as described above. Next, qPCR was performed using the QuantiTect SYBR Green PCR Kit (Fisher Scientific, NH, USA) in combination with a Stratagene Mx3005P qPCR system (Agilent Technologies, CA, USA). The effect of *Urn8* depletion and DH37 hormone stimulation on *Nha1* expression was also assessed. This was done by injecting (25 nl volume) either PBS or PBS containing DH37 peptide corresponding to a final peptide concentration of approx. $10^{-7}$M into adult animals with samples collected at 3 hours and 6 hours after treatment. Expression levels were normalized against the housekeeping gene *rp49*. All primers used are listed in *SI appendix* Table S2.

**Production of dsRNA and RNAi-mediated Knockdown.** To silence target gene expression by RNAi, transcript sequences covering app. 200-500 bp were selected. Total RNA was then extracted from rectal complexes (showing highest enrichment of *Nha1*) and cDNA synthesis was

carried out as described above. Using the cDNA as template, fragments were amplified by PCR using gene-specific primers that were tagged with T7 promoter sequences at both the 3′ and 5′ ends (see *SI Appendix* Table S2). These gene-specific fragments were then cloned into the pUC19 vector individually and subsequently verified by sequencing (Eurofins, Luxembourg, L). Using the cloned vector as template, bidirectional *in vitro* transcription was carried out using the MEGAscript T7 transcription kit (ThermoFishcer, MA, USA), and the quality of the resulting dsRNA was checked by gel electrophoresis and quantified using NanoDrop. The concentration was adjusted to 2 µg/µl using injection buffer (1.4 mM NaCl, 0.07 mM $Na_2HPO_4$, 0.03 mM $KH_2PO_4$, 4 mM KCl), and a total of 500 nl dsRNA solution was injected into age-matched adults using a Nanoject II injector (Drummond Scientific, PA, USA). Animals were allowed to recover for 2 days after injection before being used for experimentation.

**Environmental stress exposure.** In control (fed) conditions, beetles were housed individually in a 96-well plate with standard wholemeal flour containing 5% yeast. For drinking-only (water) treatments, animals were kept in 96-well plates with a small block of 1% agar with 0.05% bromophenol blue (BPB). Drinking was verified by the presence of blue deposits. For desiccation treatments, animals were kept in 96-well plates with a piece of filter paper without any nutritional and water sources at 30ºC, with individual plates kept at 5%, 50% or 90% RH.

**Desiccation tolerance.** Animals were kept on *Tribolium* medium for 3 days after dsRNA injection. Healthy animals were then transferred to a 96-well plate in a container filled with silica gel beads (Sigma-Aldrich, MO, USA) to produce a low-humidity environment (approx. RH 5%, measured by a custom-build hygrometer). The number of dead animals (not responding to tactile stimuli) were then counted every 8 h for 7 days. Data were expressed as percent survival over time.

**Hemolymph collection and quantification.** Hemolymph was collected according to the protocol described in (6, 13, 14) from animals exposed to the different environmental stress exposures as described above. In brief, animals were washed and subsequently dried on tissue paper for 2 hours to remove moisture. Then, beetles were anesthetized by $CO_2$ and their cuticle pierced between the pronotum and elytron before being transferred to an ice-cold 0.5-ml tube with a small hole in the bottom in groups of 10. This tube was then placed in a larger 1.5-ml collecting tube, which was centrifuged at 12,000 x *g* for 15 min at 4°C. Hemolymph from separate tubes were combined into each collecting tubes (containing 500 µl paraffin oil to prevent oxygen-induced melanization) from each environmental condition. Following sample collection, each sample was diluted to a final volume of 50 µl with ddH2O and the osmotic pressure of each sample was measured in triplicates on VAPRO Vapor Pressure Osmometer Model 5600 (Wescor Inc., UT, USA) with each measurement corrected according to the dilution factor of the sample.

**Quantification of water content.** To measure changes in total water content, individual beetles were transferred to a small plastic container and then measured on a Sartorius SE2 ultra micro balance (= $W_T$; Sartorius, Göttingen, DE; 0.1 µg readability). The animals were then housed under low humidity conditions as described above, and after 48 h the beetles were reweighted (= $W_{48}$). To measure the corresponding dry weight of the animals, they were kept at –20°C over night and then placed in a 65°C incubator for at least 2 days before being weighed a final time (=$W_{dry}$). The percent water loss of total body water for each animal was calculated as ($W_T - W_{T48}$)/($W_T - W_{dry}$) × 100%, with *N*=25 animals weighed for each experimental group.

**Defecation Behavior.** To assess the effects of manipulating *Nha1* expression on whole-animal excretory behavior *in vivo*, dsRNA-injected animals were starved for 2 days followed by refeeding a standard *Tribolium* medium supplemented with 0.05% (w/w) Bromophenol blue (BPB) sodium salt (Sigma-Aldrich, MO, USA) overnight as in (13). This special medium was created by mixing the standard *Tribolium* medium with BPB and a small amount of water hereby creating a uniform paste, which was left to dry at room temperature overnight. The dried BPB-labelled *Tribolium* medium was then ground to a fine powder creating a consistency identical to that of the standard medium. Beetles were then placed in individual wells of a 96-well plate fitted with a small piece of

filter paper and the number of BPB-labelled deposits produced by each animal over a 4 h period was quantified.

***Ex-vivo* fluid reabsorption assay.** The water reabsorption rate from the CNC *ex vivo* was assessed using a modified protocol (15). The control and *Nha1*-KD animals were dissected carefully, keeping the head, gut, tubules and CNC intact under Schneider's medium. MTs were then broken from the entry point (common trunk) to the CNC. The dissected animals were carefully transferred to another dish containing paraffin oil (molecular grade) with a wax layer at the bottom, and gently stretched (to keep intact the fore-, mid-, hind- gut, MTs, and CNC) and pinned at the head and anal cuticle. Two drops (5μl and 0.3μl) of premixed 3x *Tribolium* saline + Schneider's solution (1:1) supplemented with 100 μmol $l^{-1}$ of amaranth (Sigma-Aldrich, St Louis, MO, USA) were dropped under paraffin oil and their circumference measured by formula using the eye-piece graticule (10 mm = 200 parts) (Leica Microsystems, Germany). A 5μl drop was pulled around the midgut portion and MTs and a 0.3μl drop onto the CNC with the help of a capillary pull glass rod, avoiding contact with the head and cuticle. MTs were gently drawn out from the saline drop and then the open ends of MTs were wrapped around the pin with the help of fine forceps. This was left for two hours—the maximum time the system remained stable. The change in circumference of the initial and final drop was measured as $V = (\pi \times d^3)/6$, and the rate of absorbance was calculated by the following formula: $J_{fluid} = \Delta v/\Delta t$ where $J_{fluid}$ is the fluid reabsorption rate (nl $min^{-1}$), Δv is the change in volume (nl), and Δt is the duration of the experiment (min).

**Water vapor absorption assay.** The ability to extract water vapor directly from the atmosphere was quantified gravimetrically as described in (16). In brief, animals were desiccated for 2 days before being weighed and individually housed in a 96-well plate without food or water. The plate was then placed in a high-humidity chamber (RH >95%) in a temperature-controlled incubator and the weight of each animal recorded once a day for 3-6 consecutive days (depending on species) after which the animals were sacrificed, dried for 2 days at 60°C, and the reweighed to calculate the changes in body water over the time, as described above.

**Statistics.** The statistical analyses were performed using the data analysis software GraphPad Prism 9 (CA, USA). The normal (Gaussian) distribution of data were tested using the D-Agostino-Pearsen omnibus normality test. Data were plotted as mean ± SEM, Tukey's box-and-whisker plots or as raincloud plots, as indicated in each figure legend. Statistical differences between one control group and another group (unpaired samples) or between the same groups at different time points (paired samples) were compared using the two-tailed Student *t*-test, whereas differences between one control group and several other groups were pairwise compared by one-way ANOVA followed by Dunnett's multiple comparisons tests taking P=0.05 (two-tailed) as the critical value. P-values are indicated as: * $P < 0.05$, ** $P < 0.01$, *** $P < 0.001$, **** $P < 0.0001$.

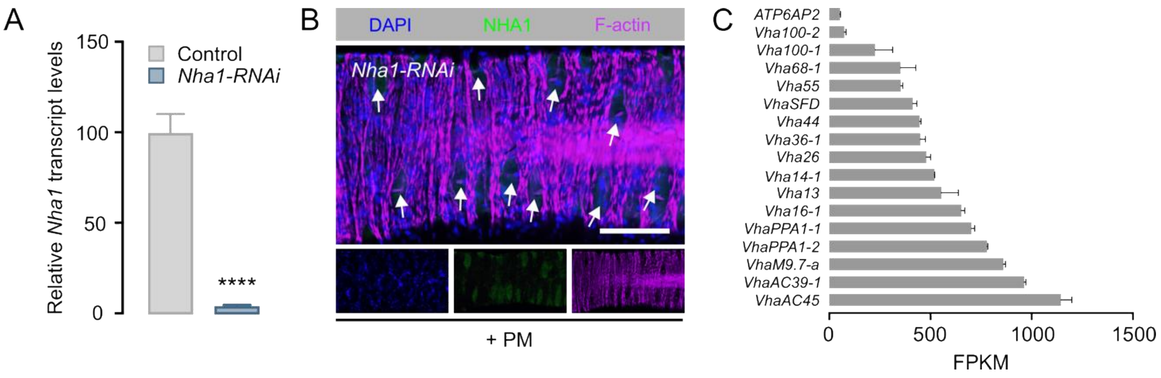


**Fig. S1.** Validation of *Nha1* knockdown efficacy. *(A) Nha1* transcript levels in the rectal complex (*n* = 5) from animals injected with dsRNA targeting the *Nha1* gene (*Nha1-RNAi*) show a significant knockdown of Nha1 expression relative to mock inject controls (unpaired Student's *t* test, **** *P*<0.0001). *(B)* Rectal complexes dissected from *Nha1*-depleted animals show almost complete depletion of anti-Nha1 immunoreactivity (arrows). (Scale bar, 100 µm). PM, perinephric membrane. *(C)* Transcript levels of V-ATPase subunit genes in the rectal complex obtained from BeetleAtlas.org.

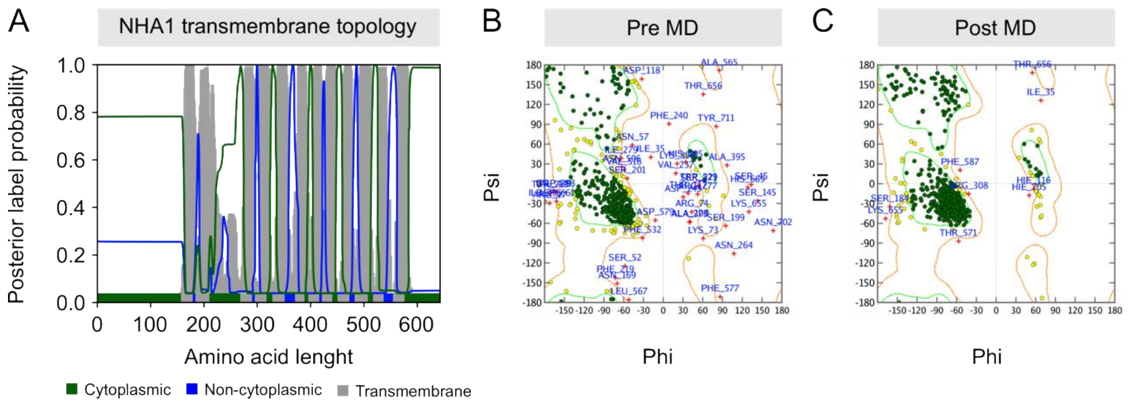


**Fig. S2.** Structural analyses of NHA1 protein. *(A)* Transmembrane topology of NHA1 revealing an 11-transmembrane domain with a tertiary structure, characteristic of other CPA2 family members. *(B)* Ramachandran plots of pre-molecular dynamics and *(C)* post-molecular dynamics (MD) simulation analyses of NHA1. The post-MD modelled structure shows an improved refinement and accuracy relative to the pre-MD structure, thus validating the use of the post-MD structure for stability analyses.

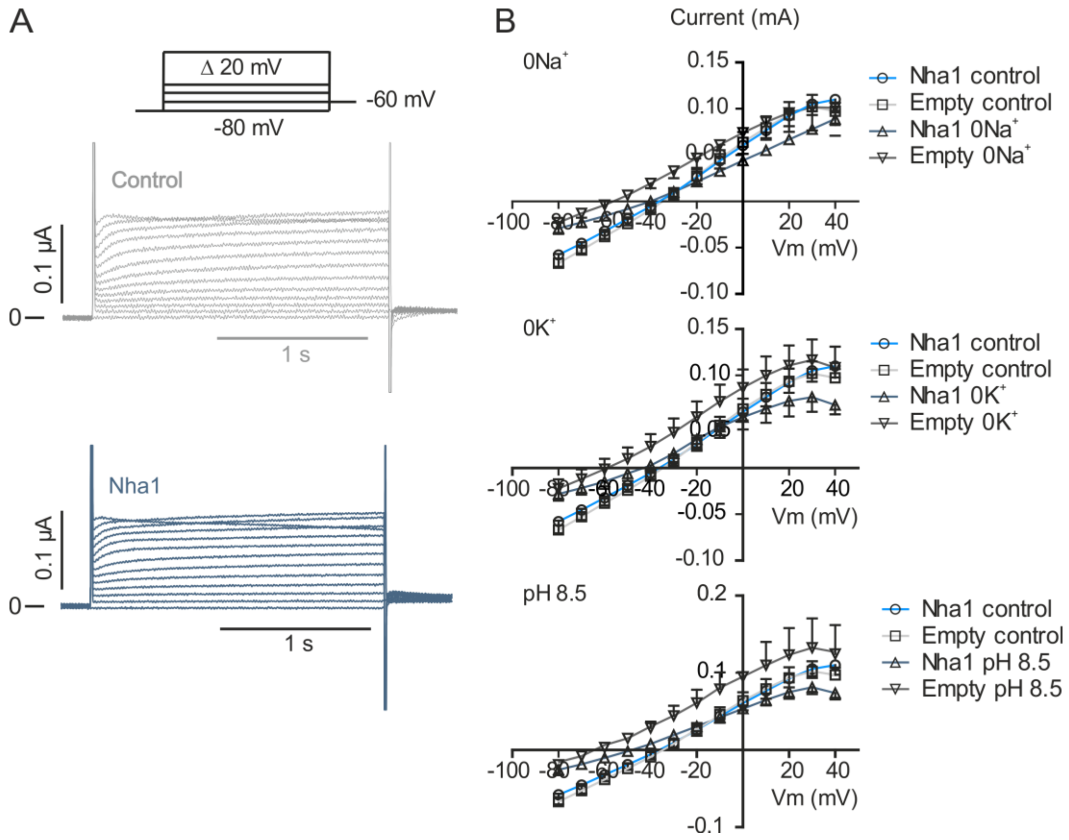


**Fig.S3.** NHA1 is electroneutral and does not exhibit voltage-dependence. *Nha1* was expressed in *Xenopus laevis* oocytes and compared to uninjected oocytes (Empty control). Currents activated by the depicted voltage-clamp protocol in Kulori's solution (control), in 0 mM $K^+$, 0 mM $Na^+$ and in pH 8.5. *(A)* Voltage-clamp protocol and representative currents recorded from empty controls and *Nha1* injected oocytes. *(B)* Mean currents plotted as a function of voltage, n=6-8.

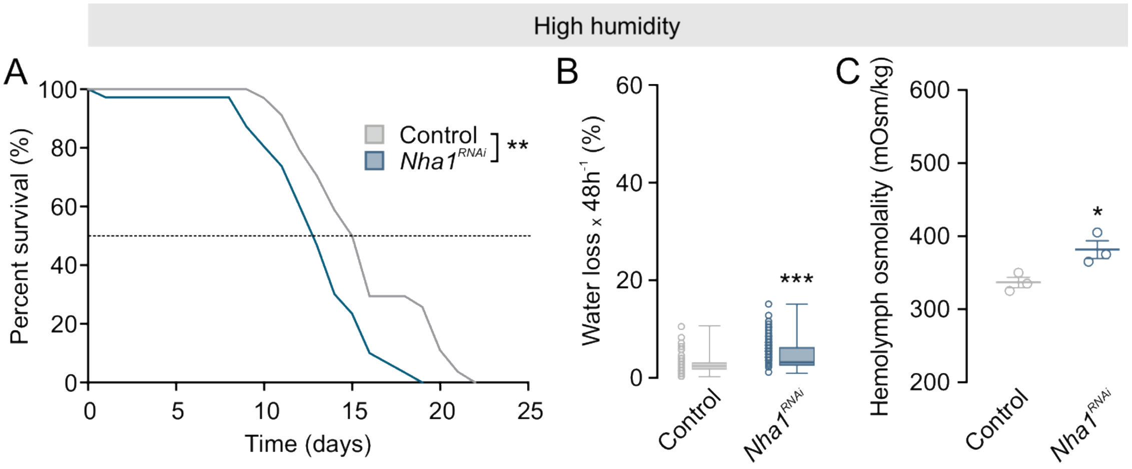


**Fig.S4. *Nha1* depletion affects systemic water balance during high humidity exposure** *(A)* Kaplan-Meyer survival function of control and *Nha1* silenced animals. Knockdown of *Nha1* reduces organismal survival when exposed to higher humidity compared to the control (RH 90%, log-rank test, $n$ = 39-44). *(B)* Gravimetric analysis of control and *Nha1*-silenced animals. Water loss is significantly increased in *Nha1* knockdown animals relative to control at high humidity (unpaired Student's $t$ test, $n$ = 57, *** $P$<0.001). *(C)* Hemolymph osmotic pressure of control and *Nha1*-depleted beetles. Hemolymph osmolality is significantly increased in *Nha1* knockdown animals relative to controls at high humidity (unpaired Student's $t$ test, $n$ = 3, *** $P$<0.05).

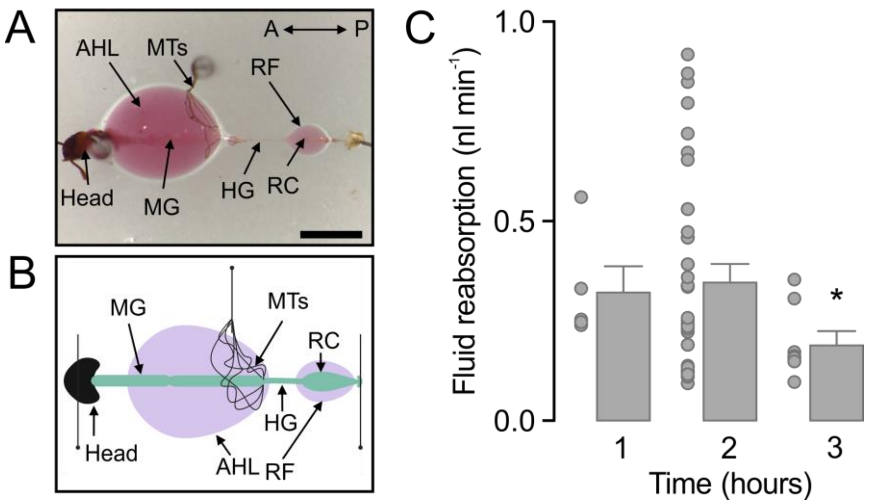


**Fig.S5.** Modified *ex vivo* fluid reabsorption assay. *(A)* Light-micrograph and *(B)* schematic representation of the *ex vivo* fluid reabsorption assay of the rectal complex. *(C)* Rate of reabsorption of fluid by the rectal complex with time. The rate of reabsorption of fluid is significantly reduced after 3 hours compared to the rate measured after 1 hour (One-way ANOVA, $n$ = 17-33, * $P$<0.05). AHL, artificial hemolymph solution; HG, hindgut; MTs, Malpighian tubules; MG, midgut; RC, rectal complex; RF, rectal reabsorbed fluid. Scale bar 0.6 mm.

**Table S1.** Examples of genes showing highly tissue-specific expression patterns according to BeetleAtlas, thus validating the specificity and quality of the RNAseq data. These data are further independently validated by qRT-PCR. Boldface indicates the maximum signal for each gene.

| | | Mean signal strength (FPKM) / qRT-PCR ($\Delta\Delta C_t$) | | | | | | | |
|---|---|---|---|---|---|---|---|---|---|
| Gene ID | Gene Symbol | Brain | | Post. Midgut | | Tubule | | Rectal complex | |
| TC030001 | Akh2 | **899** | **(100%)** | 0 | (1%) | 0 | (0%) | 1.4 | (0%) |
| TC009624 | Cht2 | 0 | (0%) | **6354** | (**100**%) | 1.8 | (1%) | 25 | (0%) |
| TC034462 | Urn8R | 9.6 | (21%) | 0.7 | (1%) | **224** | (**100**%) | 12 | (11%) |
| TC013096 | Nha1 | 17 | (24%) | 0.9 | (1%) | 0.1 | (0%) | **192** | (**100**%) |

**Table S2.** Primer sequences used for In-Fusion cloning, RT-qPCR and dsRNA synthesis. Sequences marked in red correspond to the vector sequence for In-Fusion cloning primers and to the T7 promoter sequence for dsRNA primers.

| **Cloning primer** | **Sequence** |
|---|---|
| *Tcas_ Nha1_F* | 5'-CCGGGGATCCGAATTATGTCAGTGGACCAAGATCCG-3' |
| *Tcas_ Nha1_R* | 5'-TTGCTCTAGAGAATTTTATGTTTCGCCCTTGGGC-3' |
| *Tcas_Nha1_int_F* | 5'-ATGCTCTTCACCGGGATGCTGTTG-3' |
| **qRT-PCR primer** | **Sequence** |
| *Tcas_Rp49_ F* | 5'-GTCTGACCGTTATGGCAAACTC-3' |
| *Tcas_Rp49_R* | 5'-TGTGCTTCGTTTTGGCATTGGAG-3' |
| *Tcas_Nha1_F* | 5'-TTGCGTTAGCGTGGATGTCAAAGG-3' |
| *Tcas_Nha1_R* | 5'-ATTTAGTCTTGGTGAGCAGCCGAG-3 |
| *Tcas_Akh_F* | 5'-ATCCCGTTGAGAAAATGCATCG-3' |
| *Tcas_Akh_R* | 5'-TCCACTAATTTCTGCGCCTCGTTC-3' |
| *Tcas_Cth2_F* | 5'-ATTTCAGCGGTGCCCTCGTTTC-3' |
| *Tcas_Cth2_R* | 5'-CCACGTCCAAATACTCATCCAAG-3' |
| *Tcas_Urn8-R_F* | 5'-TTATCACAATCTACGCCCCAACCCC-3' |
| *Tcas_Urn8-R_R* | 5'-AATAGAGCGACAGTGAAGCCCTGTG-3' |
| *Tcas_Urn8_F* | 5'-TTATCACAATCTACGCCCCAACCCC-3' |
| *Tcas_Urn8_R* | 5'-AATAGAGCGACAGTGAAGCCCTGTG-3' |
| **dsRNA primer** | **Sequence** |
| *T7_Amp_R* | 5'-TAATACGACTCACTATAGGTTACCAATGCTTAATCAGTGAGGCACC-3' |
| *T7_Amp_F* | 5'-TAATACGACTCACTATAGGATGAGTATTCAACATTTCCGTGTCGCCC-3' |
| *T7_Nha1_R* | 5'-TAATACGACTCACTATAGGGAGATCCAAGCACAGAAATCGGCCC-3' |
| *T7_Nha1_F* | 5'-TAATACGACTCACTATAGGGAGAATGTTGCCCTCGTGATTATCC-3' |
| *T7_Urn8_F* | 5'-TAATACGACTCACTATAGGATGTGCCATCGTTTCGCCAAAACTG-3' |
| *T7_Urn8_R* | 5'-TAATACGACTCACTATAGGCTGATTTTCGATGTCCTGCGATTCC-3' |

**SI Movie S1.** Three-dimensional tertiary structure of NHA1 as predicted by I-TASSER and subsequent Ramachandran plot analysis for structural refinement with putative $Na^+$ and $K^+$ binding sites as predicted by molecular dynamics simulation using the AMBER suite on the Computerome 2.0 high-performance computer cluster.

## SI References


1. K. A. Halberg, S. Terhzaz, P. Cabrero, S. A. Davies, J. A. Dow, Tracing the evolutionary origins of insect renal function. *Nat Commun* **6**, 6800 (2015).
2. C. Trapnell *et al.*, Differential gene and transcript expression analysis of RNA-seq experiments with TopHat and Cufflinks. *Nat Protoc* **7**, 562-578 (2012).
3. N. Herndon *et al.*, Enhanced genome assembly and a new official gene set for Tribolium castaneum. *BMC Genomics* **21**, 47 (2020).
4. K. A. Halberg *et al.*, The cell adhesion molecule Fasciclin2 regulates brush border length and organization in Drosophila renal tubules. *Nat Commun* **7** (2016).
5. L. Lison, Sur la structure de la region cryptosoléniée chez les coléoptères, *Tenebrio molitor* L. et *Dermestes lardius* L. . *Bull. Acad. R. Belg.* **23**, 137-327 (1937).
6. T. Koyama *et al.*, A nutrient-responsive hormonal circuit mediates an inter-tissue program regulating metabolic homeostasis in adult Drosophila. *Nature Communications* **12**, 5178 (2021).
7. G. W. Maurer *et al.*, Analysis of genes within the schizophrenia-linked 22q11.2 deletion identifies interaction of night owl/LZTR1 and NF1 in GABAergic sleep control. *PLoS Genet* **16**, e1008727 (2020).
8. K. A. Halberg, N. Møbjerg, First evidence of epithelial transport in tardigrades: a comparative investigation of organic anion transport. *J Exp Biol* **215**, 497-507 (2012).
9. E. Laugier, Z. Yang, L. Fasano, S. Kerridge, C. Vola, A critical role of teashirt for patterning the ventral epidermis is masked by ectopic expression of tiptop, a paralog of teashirt in Drosophila. *Dev Biol* **283**, 446-458 (2005).
10. J. Yang *et al.*, The I-TASSER Suite: protein structure and function prediction. *Nature Methods* **12**, 7-8 (2015).
11. L. F. Song, T.-S. Lee, C. Zhu, D. M. York, K. M. Merz, Using AMBER18 for Relative Free Energy Calculations. *Journal of Chemical Information and Modeling* **59**, 3128-3135 (2019).
12. S. Javaid *et al.*, Computational and biological characterization of fusion proteins of two insecticidal proteins for control of insect pests. *Sci Rep* **8**, 4837 (2018).
13. T. Koyama *et al.*, A unique Malpighian tubule architecture in Tribolium castaneum informs the evolutionary origins of systemic osmoregulation in beetles. *Proc Natl Acad Sci U S A* **118** (2021).
14. K. A. Halberg, K. W. Larsen, A. Jørgensen, H. Ramløv, N. Møbjerg, Inorganic ion composition in Tardigrada: cryptobionts contain a large fraction of unidentified organic solutes. *Journal of Experimental Biology* **216**, 1235-1243 (2013).
15. J. H. Tupy, J. Machin, Transport characteristics of the isolated rectal complex of the mealworm Tenebrio molitor. *Canadian Journal of Zoology* **63**, 1897-1903 (1985).
16. J. Machin, Water balance inTenebrio molitor, L. Larvae; the effect of atmospheric water absorption. *Journal of comparative physiology* **101**, 121-132 (1975).
